# Fungal Biodiversity and Their Role in Soil Health

**DOI:** 10.3389/fmicb.2018.00707

**Published:** 2018-04-13

**Authors:** Magdalena Frąc, Silja E. Hannula, Marta Bełka, Małgorzata Jędryczka

**Affiliations:** ^1^Institute of Agrophysics, Polish Academy of Sciences, Lublin, Poland; ^2^Netherlands Institute of Ecology, Wageningen, Netherlands; ^3^Department of Forest Pathology, Poznań University of Life Sciences, Poznań, Poland; ^4^Institute of Plant Genetics, Polish Academy of Sciences, Poznań, Poland

**Keywords:** soil health, soil ecosystem, microbial communities, fungal diversity, fungal functions, fungal plant pathogens, soil biology, soil mycobiome

## Abstract

Soil health, and the closely related terms of soil quality and fertility, is considered as one of the most important characteristics of soil ecosystems. The integrated approach to soil health assumes that soil is a living system and soil health results from the interaction between different processes and properties, with a strong effect on the activity of soil microbiota. All soils can be described using physical, chemical, and biological properties, but adaptation to environmental changes, driven by the processes of natural selection, are unique to the latter one. This mini review focuses on fungal biodiversity and its role in the health of managed soils as well as on the current methods used in soil mycobiome identification and utilization next generation sequencing (NGS) approaches. The authors separately focus on agriculture and horticulture as well as grassland and forest ecosystems. Moreover, this mini review describes the effect of land-use on the biodiversity and succession of fungi. In conclusion, the authors recommend a shift from cataloging fungal species in different soil ecosystems toward a more global analysis based on functions and interactions between organisms.

## Fungi in Soils

**FIGURE 1:**
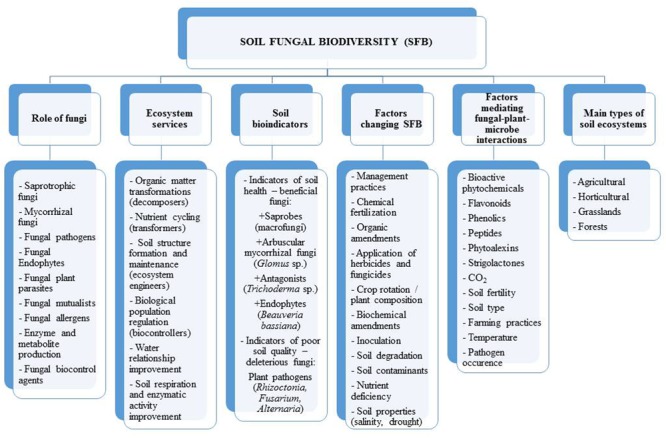
Aspects of soil fungal biodiversity.

Fungi are very successful inhabitants of soil, due to their high plasticity and their capacity to adopt various forms in response to adverse or unfavorable conditions (Sun et al., 2005). Due to their ability to produce a wide variety of extracellular enzymes, they are able to break down all kinds of organic matter, decomposing soil components and thereby regulating the balance of carbon and nutrients (Žifčáková et al., 2016). Fungi convert dead organic matter into biomass, carbon dioxide, and organic acids (**Figure 1**). Many species of fungi possess the ability to act as an effective biosorbent of toxic metals such as cadmium, copper, mercury, lead, and zinc, by accumulating them in their fruiting bodies. Though these elements may inhibit their growth and affect their reproduction (Baldrian, 2003). The diversity and activity of fungi is regulated by various biotic (plants and other organisms) and abiotic (soil pH, moisture, salinity, structure, and temperature) factors (López-Bucio et al., 2015; Rouphael et al., 2015). Fungi can be found in almost every environment and can live in wide range of pH and temperature (Frąc et al., 2015).

Soil fungi can be classified into three functional groups including: (1) biological controllers, (2) ecosystem regulators, and (3) species participating in organic matter decomposition and compound transformations (Swift, 2005; Gardi and Jeffery, 2009). Ecosystem regulators are responsible for soil structure formation and modification of habitats for other organisms by regulating the dynamics of physiological processes in the soil environment. Biological controllers can regulate diseases, pests, and the growth of other organisms (Bagyaraj and Ashwin, 2017). For example, the mycorrhizal fungi improve plant growth by increasing the uptake of nutrients and protect them against pathogens (Bagyaraj and Ashwin, 2017).

Fungal populations are strongly influenced by the diversity and composition of the plant community and in return affect plant growth through mutualism, pathogenicity and their effect on nutrient availability and cycling (Wardle, 2002; Wagg et al., 2014; Hannula et al., 2017). Moreover, fungi participate in nitrogen fixation, hormone production, biological control against root pathogens and protection against drought (Jayne and Quigley, 2014; Baum et al., 2015; El-Komy et al., 2015). They also play an important role in stabilization of soil organic matter and decomposition of residues (Treseder and Lennon, 2015).

## Methods and Recent Achievements in Studies of Soil-Borne Fungi

The advent of next generation sequencing (NGS) has facilitated a sea-change in the analysis of soil and plant-associated fungal communities. Standardized pipelines for preparing rhizosphere soil samples for Illumina sequencing are widely available (Lindahl et al., 2013; Schöler et al., 2017) and in a relatively short time following sampling, files with millions of sequences can be generated. Important points to consider when preparing samples for NGS are: sufficient biological replication (Prosser, 2010), sufficient sequencing depth (Smith and Peay, 2014; Weiss et al., 2015), adequate coverage of target organisms (i.e., primer selection and DNA extraction), and the avoidance of contamination and bias (Salter et al., 2014; Schöler et al., 2017). There are multiple pipelines for the analysis of fungal NGS data available (Bálint et al., 2014; Gweon et al., 2015), so it is not data-analysis that is problematic, but the interpretation of results.

Alpha-diversity, representing either the number of species or diversity indices that account for evenness, was proposed as an indicator for robust, healthy soil (Ferris and Tuomisto, 2015). However, it is open to question whether absolute diversity or functional diversity should be emphasized (Wagg et al., 2014; Ferris and Tuomisto, 2015). Usually the second step in analysis is to look at beta-diversity to see the effects of treatment/manipulation on the fungal community. Recently, it has been shown that fungal biodiversity in soils is strongly affected by plant community (Yang et al., 2017), soil moisture (Bagyaraj and Ashwin, 2017), and the intensity of agricultural practices (Thomson et al., 2015). Studies identified key fungal species affected by soil treatments, but it is unknown if results obtained from studies conducted on one particular soil and ecosystem can be used to infer trends and identify key fungal groups at a global or continental scale (Tedersoo et al., 2014; Delgado-Baquerizo et al., 2017).

Unlike the analysis of bacteria and archaea, where 16S rRNA is used as a barcode, fungi are usually identified based on the sequence of the Internal Transcribed Spacer (ITS) region allowing identification up to species level (Schoch et al., 2012; Porras-Alfaro et al., 2014). Although in some cases, such as for soil-dwelling Fusaria, sequencing of additional genes, such as β-tubulin gene (β*-Tub*), and aminoadipate reductase gene (*LYS2*) was proposed to obtain the correct taxonomic identification at the species level (Watanabe et al., 2011), whereas others have suggested the use of Translation Elongation Factor (Geiser et al., 2004). Many researchers have in-house databases for functional classification of fungi but recently online resources for functional annotation of fungi have been made publicly available (Nguyen et al., 2016).

**FIGURE 2:**
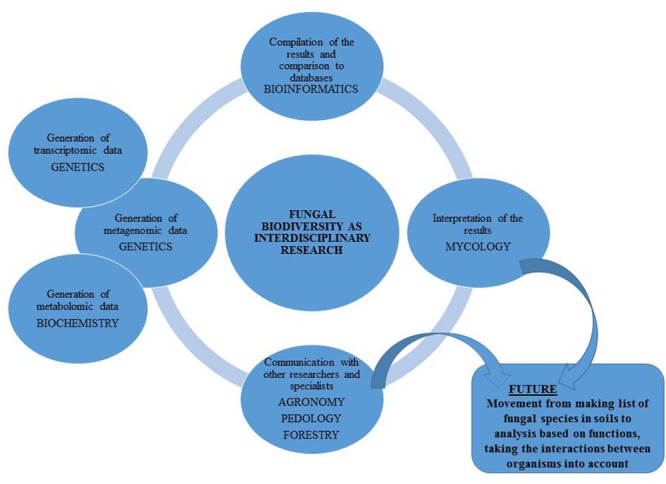
Soil fungal biodiversity as an interdisciplinary research.

Species level identification is, however only the first step from ‘What is there?’ toward the question ‘What role does it play?’ (**Figure 2**). Identification neither implies the microorganisms are alive and active (Blagodatskaya and Kuzyakov, 2013) nor does it describe their function (Prosser, 2015). To unravel the function of the community, either (shotgun) metagenomics (Uroz et al., 2013; Hannula and van Veen, 2016; Castañeda and Barbosa, 2017), metatranscriptomics (Damon et al., 2012; Turner et al., 2013; Hesse et al., 2015) or time-intensive culture based methods combined with functional tests must be used (Behnke-Borowczyk et al., 2012; Gałązka et al., 2017; Wolińska et al., 2017). A fast, but coarse alternative to molecular methods is MicroResp (Creamer et al., 2016) or community level physiological profiles (CLPP) approach (Frąc et al., 2017), which gives an indication on substrate use of the total microbial community. This method, however, does not identify the species responsible for the process. Increasingly, especially in studies where plant community is included alongside NGS approaches, microorganisms are isolated from the soils and plant roots for further functional testing.

To construct a more complete picture of a soil fungi community their interactions with other organisms must be taken into consideration. Strong linkage was proved between functional soil biodiversity and the function of the soil ecosystem (Wagg et al., 2014; Delgado-Baquerizo et al., 2017; Morrien et al., 2017). Fungi interact with other soil organisms and thus changes in the fungal community have the potential to affect the function of the whole soil ecosystem (Yang et al., 2017). Analysis of the interactions in the soil can be achieved through indirect estimation of species interactions using co-occurrence networks (Creamer et al., 2016; Morrien et al., 2017) or directly by using isotope tracers (Hannula et al., 2017) and/or gut content analysis (Kurakov et al., 2016).

## Fungal Biodiversity and Their Functions in Soil Health of Agricultural and Horticultural Ecosystems

The term ‘soil health’ is widely used in reference to sustainable agriculture (Kibblewhite et al., 2008; Cardoso et al., 2013), especially in the context of soil as a dynamic, living organism functioning holistically rather than as an inert substrate (Doran and Jones, 1996). Therefore, in this article we prefer to use the terminology of soil health, rather than soil quality, which is defined as the capacity of the soil to maintain environmental quality, sustain biological productivity, and promote animal, human, and plant health (Doran and Parkin, 1994). In recent years the potential application of cultivating soil fungal biodiversity to improve soil quality and increase productivity of agricultural ecosystems has been highlighted as a new and very promising development in plant productivity (Bagyaraj and Ashwin, 2017), which may come be called ‘the 2nd Green Revolution.’ The implementation of such solutions may offer an alternative to the current overuse of fertilizers toward more sophisticated manipulations of plant productivity. Fungi participate in decomposition of organic matter and deliver nutrients for plant growth. Their role is very important in plant protection against pathogenic microorganisms as biological agents, which influences soil health (Frąc et al., 2015). The assessment of fungal biodiversity as quality indicators cannot be limited only to the determination of biodiversity indexes, but also should include a structure analysis of fungal population in order to determine the functions they play in affecting soil quality and plant health. The use of different kinds of organic manure has a strong influence on soil health, through indirect effects (i.e., via changes in physicochemical characteristics) and a direct effect on soil fungal communities. Soil management is fundamental to all agricultural systems, and the reduction of soil degradation is a priority to sustain future production. This effect can be only achieved by taking soil fungal biodiversity into account. All cultural practices, such as the use of cover and rotational crops, composts and tillage systems, besides their known effects on soil-borne pathogens (Abawi and Widmer, 2000), are likely to affect also the other groups of soil fungi, especially beneficial fungal populations. It has long been known that the suppressiveness of soils can be enhanced by adding biopolymers such as chitin and its derivatives (i.e., chitosan). This suppressiveness is related to a change in the activity and structure of soil microorganisms (Cretoiu et al., 2013). Therefore, we should utilize our knowledge on the interactions between different fungal groups and their ecology in the management of agricultural systems. It is worth mentioning that chitin addition to the soil increases bacteria and fungi that can degrade pathogenic fungal cell walls and can thus, increase the soil suppressiveness against plant pathogens. This might be a good alternative to fungicides that kill all fungi, including beneficial ones. Different tillage treatments can also impact soil fungi by soil disturbances that affect the functioning of fungal communities. Reduced tillage decreases the breakdown of hyphae causing fungal populations to remain more stable, retaining more nutrients and providing more suppressive effects against pathogenic microorganisms (Goss and deVarennes, 2002). Understanding and selecting the appropriate cultural practices, increasing fungal biodiversity, can prevent or decrease damage of root diseases and play a crucial role in the maintenance of soil quality and health. It should be taken into account that fungal diversity determines plant biodiversity, ecosystem variability, and productivity (van der Heijden et al., 1998; Wagg et al., 2014).

Arbuscular mycorrhizal fungi (AMF) are the most important class of beneficial microorganisms in agri- and horticultural soils (Smith and Read, 2008, **Table 1**). Significant increases in the yield of crop plants following inoculation with AMF have been observed in numerous experiments (Thilagar and Bagyaraj, 2015; Bagyaraj and Ashwin, 2017). The key effects of AMF symbiosis include: improvement of rooting and plant establishment, stimulation of nutrient cycling, improvement of soil structure, enhancement of plant tolerance to stresses, increased uptake of low mobility ions, and enhancement of plant community diversity (Azcón-Aguilar and Barea, 1997). The diseases of crop plants can be controlled by some antagonistic fungi such as *Glomus* sp. or *Trichoderma* sp. suppressing fungal pathogens (Dawidziuk et al., 2016). Species of *Trichoderma* (*T. asperellum, T. atroviride, T. harzianum, T. virens*, and *T. viride*) are frequently used in biocontrol and are known as biostimulants for horticultural crops (López-Bucio et al., 2015). Other positive effects of fungi on soil quality and plant health include inoculation by microbial consortia of AMF together with plant growth promoting rhizobacteria (PGPR) and others such as N-fixing and P-solubilizing microorganisms (Bagyaraj and Ashwin, 2017). A synergistic, favorable impact of AMFs and PGPRs on horticultural plant growth and soil microbial diversity and activity has been reported (Azcón-Aguilar and Barea, 1997; De Coninck et al., 2015).

**Table 1:** Fungal community composition in different soil ecosystems and their function.

Ecosystem	Fungal composition	Fungal function/reaction	Reference
Agricultural	*Agaricales* *Hypocreales*	Increase in drought-affected soils	Bastida et al., 2017
	*Sordariales* *Capnodiales* *Eurotiales*	Reduction in soils affected by drought	
	*Mortierella* *Fusarium* *Gibberella*	The dominant fungi in NPK treated soils	Ding et al., 2017
	*Mortierella* *Fusarium* *Schizothecium*	The dominant fungi in manure treated soils	
	Ascomycota *Sordariomycetes* *Eurotiomycetes* *Dothideomycetes*	Key decomposers in agricultural soils. Increase after nitrogen fertilization	Žifčáková et al., 2016
	*Leotiomycetes* *Helotiales*	Decline in N and P treated soils	
	*Cyphellophora* *Penicillium* *Chloridium* *Trichoderma* *Acremonium*	Increase in fertilized soils	
	*Exophiala* *Clonostachys* *Sarocladium* *Schizothecium* *Magnaporthe* *Phaeosphaeriopsis*	Decrease in N and P fertilized soils	
Horticultural	*Glomus* *Gigaspora* *Scutellospora* *Acaulospora* *Entrophospora*	Arbuscular mycorrhizal fungi (AMF) which improve plant growth by increasing phosphorus and the uptake of other nutrients	Smith and Read, 2008; Bagyaraj and Ashwin, 2017
	*Trichoderma* such as: *T. asperellum* *T. atroviride* *T. harzianum* *T. virens* *T. viride*	Biostimulants and biocontrol agents, suppressing fungal pathogens like *Fusarium* sp., *Colletotrichum* sp., or *Rhizoctonia* sp.	López-Bucio et al., 2015
Grasslands	*Hygrocybe* *Camarophyllopsis* *Dermoloma* *Entoloma* *Clavariaceae* *Geoglossaceae*	Saprotrophs, decomposers	Arnolds, 2001; Öster, 2006; Hannula et al., 2017
	*Glomus*	Play a dominant role under different conditions of grasslands as AMF	Johnson et al., 2003; Santos-González, 2007
	*Agaricomycotina Saccharomyceta Paraglomerales* *Chytridiales* *Archaeosporales* *Lobulomycetales Rhizophydiales* *Glomerales*	AMF, pathogens or decomposers	Cassman et al., 2016
	*Acaulospora* *Glomus* *Scutellospora*	AMF important for soil and plant health, the species richness decrease in severely degraded grasslands	Cai et al., 2014
	Ascomycota Glomeromycota	Increasein Ascomycota and decrease in Glomeromycota after N and P addition into the soil. Glomeromycota phylum is composed almost entirely of AMF	Leff et al., 2015
	Hypocreales Pezizales Dothideales (*Aureobasidium*)	Most of them is known as plant pathogens	Porras-Alfaro et al., 2011
	Pleosporales such as: *Alternaria Cochliobolus/Bipolaris* *Phaeosphaeria* *Leptosphaeria* *Phoma*		
	Saprotrophic fungi	Decomposers which convert organic matter and produce enzymes	Lucas et al., 2007; Porras-Alfaro et al., 2011
Forests	*Armillaria* *Phellinus* *Cronartium* *Arceuthobium*	Pathogen of trees	Winder and Shamoun, 2006; Wakelin et al., 2014
	*Clonostachys candelabrum* *Geomyces pannorum* *Penicillium adametzii* *P. commune* *P. daleae*, *P. janczewskii* *Trichoderma*	Antagonistic microbes suppressing soil-borne plant pathogens	Pal et al., 2006; Małecka et al., 2015
	Basidiomycota Ascomycota	Ectomycorrhizal mutualists which protect plant families such as Pinaceae, Fabaceae, Betulaceae, and Fagaceae	Rillig and Mummey, 2006; Phosri et al., 2012
	Macrofungi	Biosorbents of toxic metals and compounds	Baldrian, 2003
	Tremellomycetes Dothideomycetes	The dominant fungal class in forest soil	Liu et al., 2015

Besides beneficial fungi, agri- and horticultural ecosystems contain also plant pathogens. The major groups of soil-borne root pathogenic fungi and oomycetes constitute of genera *Fusarium* (Michielse and Rep, 2009), *Verticillium* (Klosterman et al., 2009), *Rhizoctonia* (Gonzalez et al., 2011), *Pythium, Phytophthora* (van West et al., 2003) and many others, of global and local importance. The soil fungal diversity and methods of increasing it, particularly the populations of beneficial fungi within ecosystems should be used in practice for more sustainable plant production, decrease of chemical applications and protection of the soil environment.

## Fungal Biodiversity and Their Functions in Soil Health of Grassland Ecosystems

Soil microorganisms, including fungi are an important component of grassland ecosystems due to their biochemical activity and engagement in nutrient cycling (Dengler et al., 2014). Grasslands provide many forms of ecosystem services including: supporting, provisioning, regulatory, and cultural services. Importantly, the role of biodiversity has been established as fundamental in ensuring the performance of ecosystem functioning. Grazing activities influence soil fungal community structure by changing edaphic conditions and the vegetation biodiversity in plant communities (Yang et al., 2017). It has been proven that moderate grazing sustains plants diversity while heavy grazing results in species loss (Joubert et al., 2017). Furthermore, plant-fungal interactions can inhibit biodiversity in grasslands due to the production of different root exudates such as enzymes, organic compounds, and polysaccharides (Huhe et al., 2017).

Plant pathogenic fungi also have a large impact on plant diversity in grasslands by limiting the abundance of their hosts, affecting biomass production. The study by Allan et al. (2010) suggests that fungal pathogens could affect nutrient cycling in grasslands reducing the abundance of dominant grasses and enhancing the growth of legumes. Soil fungal communities in grasslands can also be influenced by human activities and the components of long-term fertilization and other treatments (Cassman et al., 2016). Unlike in agricultural soils, where ascomycetes dominate, in grasslands, basidiomycetes are major decomposers of dead organic matter (Deacon et al., 2006).

## Fungal Biodiversity and Their Functions in Soil Health of Forest Ecosystems

Knowledge of the soil chemical and physical properties has always been of interest to foresters to evaluate the capacity of sites and to increase forest productivity (Schoenholtz et al., 2000). Forest soils (including humus, litter, and coarse woody debris) are an important reservoir of microorganisms and soil biota that in turn influence carbon storage, soil structure, fertility, productivity, and plant/tree growth.

Ectomycorrhizal associations are created by a specific group of plant families that includes the Pinaceae, Fabaceae, Betulaceae, and Fagaceae (Phosri et al., 2012). The results of research obtained by Högberg and Högberg (2002), indicate a significant contribution by ectomycorrhizal mycelium to forest soil microbial biomass and by ectomycorrhizal roots to the production of extractable dissolved organic carbon, which is a carbon source for other microbes.

During the processes of thinning, the transfer of nutrients from aboveground biomass to forest soil takes place (Tian et al., 2010). A higher concentration of nutrients comes from the green litter of thinned trees than litter returned to the forest floor after senescence (Girisha et al., 2003) or from the woody residue left on the ground after harvesting (Cookson et al., 2008). Consequently, the quality and quantity of organic substrates presented to the soil fungal community by thinned and non-thinned forests may vary to a great extent. The community of soil microorganisms depends highly on organic matter as it provides a suitable environment and energy sources for them that are critical to maintain the nutritional quality and water-retaining capacity of forest soils (Jiménez-Morillo et al., 2016). Soil organic matter is of key relevance in maintaining soil resistance and stability, although it is uncertain how deterioration of soil properties and changes in fungal communities affect the functional stability of soils. Degradation of soil properties followed by deforestation may lead to decreases in soil fungal diversity and functional stability (Chaer et al., 2009).

## Concluding Remarks

Soil health conditions have a tremendous impact on environmental sustainability including sustainability in agriculture, horticulture, and forestry. Moreover, soil health is directly connected with the production of healthy food which impacts public and animal health. More research is required to find the best way to maintain fungal biodiversity in soil, taking into consideration fungal functions and ecosystem services, including disease control, contamination detection, and bioremediation. Having the right tools, and being able to both identify species and characterize their role in the environment is important. The ability to compare functional structures between ecosystems and predict responses to environmental changes and interventions would be a useful advance.

## Author Contributions

MF, SH, MB, and MJ: wrote, drafted, read, corrected, improved, revised, and accepted the last version of manuscript.

## Conflict of Interest Statement

The authors declare that the research was conducted in the absence of any commercial or financial relationships that could be construed as a potential conflict of interest.
